# The role of oxidative balance lifestyle factors in reducing female infertility risk: insights from a population-based study

**DOI:** 10.3389/fendo.2025.1444832

**Published:** 2025-06-19

**Authors:** Ping Xia, Xiaobao Chen, Rong Lin, Xiaolong Shi, Yunling Yang, Liang Lin

**Affiliations:** ^1^ Medical Center of Maternity and Child Health, Shengli Clinical Medical College of Fujian Medical University, Fuzhou University Affiliated Provincial Hospital, Fuzhou, China; ^2^ Department of Urology, Fujian Medical University Union Hospital, Fuzhou, China

**Keywords:** oxidative balance score, infertility, antioxidant, lifestyle, NHANES

## Abstract

**Background:**

Diet, lifestyle, and oxidative stress have been linked to female infertility, with the Oxidative Balance Score (OBS) serving as a comprehensive indicator of an individual’s oxidative and antioxidant status. This study aims to investigate the correlation between OBS and female infertility.

**Methods:**

The National Health and Nutrition Examination Survey (NHANES) from 2013 to 2020 were utilized. Weighted multivariate regression analyses and restricted cubic splines (RCS) were employed to analysis. Additionally, subgroup analyses and multiple imputat6ions (MI) were carried out as sensitivity analyses to ensure the strength and reliability of the findings.

**Results:**

A total of 3,905 individuals were involved in the study, the prevalence of female infertility was 11.96%. Individuals with infertility exhibited significantly lower OBS compared to those with normal fertility (19.74 ± 0.37 vs. 21.42 ± 0.20). The OBS dietary and lifestyle components also had lower scores, with averages of 15.98 ± 0.33 vs. 17.12 ± 0.18 and 3.76 ± 0.11 vs. 4.29 ± 0.05, respectively. Weighted logistic regression results revealed that a one-point increase in OBS score was associated with a 3% decrease in infertility risk (Odds Ratio (OR) 0.97, 95% Confidence Interval (CI): 0.95, 0.99). Similarly, a one-point increase in OBS lifestyle score was linked to a 15% decrease in infertility risk (OR: 0.85, 95% CI: 0.75, 0.96), and a one-point increase in OBS dietary score was associated with a 2% decrease in infertility risk (OR: 0.98, 95% CI: 0.96, 0.99). Subgroup analyses revealed that individuals with no prior history of pregnancy benefited more from OBS and OBS lifestyle in terms of infertility risk reduction compared to those with a history of pregnancy.

**Conclusion:**

OBS is found to have a negative correlation with infertility, particularly in cases of primary infertility. The results of this study indicate that adopting an antioxidant-rich diet and lifestyle could potentially lower the risk of infertility.

## Introduction

Infertility, defined as the inability to conceive after at least 12 months of consistent, unprotected sexual activity (1) affects approximately 15% of couples worldwide during their reproductive years. This rate is on a steadily increasing year by year. Previous studies (2, 3) on the global burden of infertility from 1990 to 2017 showed a 0.37% yearly rise in the age-standardized prevalence of infertility. Beyond its direct impact on reproductive health, female infertility has been identified as a potential risk factor for the development of cancers in the reproductive system and metabolic disorders (4, 5). This escalating prevalence, coupled with its broad-ranging implications, underscores the urgent need to address infertility as a critical global health issue.

The etiology of female infertility encompasses multiple factors, including ovulatory dysfunction, fallopian tube pathology, endometriosis, uterine abnormalities, and unexplained causes (6, 7). While medical conditions contribute significantly to infertility, modifiable lifestyle and environmental factors are increasingly recognized for their role. Many of these factors exert their effects through oxidative stress pathways, which may present opportunities for targeted intervention.

Oxidative stress, characterized by an imbalance between reactive oxygen species (ROS) production and the body’s antioxidant defense mechanisms, is recognized as a crucial factor in the development of various chronic diseases, including cardiovascular disease, type 2 diabetes, chronic kidney disease, and osteoarthritis (8–12). To assess this complex interplay, the Oxidative Balance Score (OBS) was developed (13, 14). The OBS is a comprehensive measure that integrates dietary and lifestyle factors to evaluate an individual’s oxidative and antioxidant status. Unlike individual biomarkers, the OBS provides a holistic assessment by incorporating both pro-oxidant and antioxidant dietary components, alongside lifestyle factors such as physical activity, body mass index (BMI), alcohol consumption, and smoking status. The OBS is calculated based on a weighted combination of these factors, with specific weights assigned to each component according to its relative contribution to oxidative stress. Over time, the OBS has been refined to enhance its predictive accuracy and applicability across diverse populations, emphasizing its growing importance as an epidemiological tool.

Additionally, the OBS offers valuable insights into the potential benefits of lifestyle modifications in reducing oxidative stress. Our study aims to fill this gap by providing a comprehensive evaluation of the relationship between OBS and female infertility.

Although previous studies (15–17) have examined isolated aspects of oxidative stress in relation to fertility, the association between comprehensive oxidative balance and female infertility remains largely unexplored. We hypothesize that a higher OBS, indicative of better oxidative balance, is associated with a lower risk of infertility, potentially with differential effects based on age and pregnancy history. This hypothesis is supported by the known age-related changes in oxidative stress and the varying etiologies of infertility across different age groups. This population-based study leverages NHANES data to investigate the relationship between OBS and female infertility, with a particular focus on examining variations across different subgroups of women. Our findings may have significant clinical implications, suggesting that targeted lifestyle interventions aimed at improving oxidative balance could be an effective strategy for reducing the risk of infertility.

## Methods

### Study population

This cross-sectional study leveraged data from the NHANES, conducted between 2013 and 2020. The NHANES, managed by the National Center for Health Statistics (NCHS), is carried out every two years to assess the health and nutritional status of the non-institutionalized civilian population in the United States. Using a complex, multistage stratified sampling approach, NHANES collects data from a diverse group of participants through surveys, physical exams, and lab tests, offering a thorough and scientifically sound evaluation of public health. The initial screening encompassed 15,689 participants. After excluding females outside the age range of 20 to 45 years (n=10,380), those without reproductive health data (n=223), and participants lacking obstetric service information (n=1,181), the final study population comprised 3,905 women.

### Measurement of OBS

The OBS is derived from a comprehensive assessment that integrates 16 dietary components and 4 lifestyle elements, encompassing 15 antioxidants and 5 pro-oxidants (13, 14). **Supplementary Table 1** illustrates the detailed scoring system for OBS. Dietary antioxidants are scored from 0 to 2, with the highest tertile receiving a score of 0 and the lowest tertile a score of 2 for pro-oxidants. Lifestyle factors were assessed using a standardized scoring system: individuals received 0 points for engaging in less than 400 MET-minutes per week, 1 point for 400 to 1,000 MET-minutes, and 2 points for exceeding 1,000 MET-minutes weekly in terms of physical activity. For alcohol consumption, a score of 0 was assigned for intakes above 30 g/day, 1 point for 0 to 30 g/day, and 2 points for abstainers. Body Mass Index (BMI) was categorized as 0 points for obesity, 1 point for overweight, and 2 points for normal weight individuals. Serum cotinine levels were scored as 0 points for concentrations above 0.038 ng/mL, 1 point for levels between 0.038 to 1.13 ng/mL, and 2 points for values below 1.13 ng/mL. This systematic scoring system facilitated the evaluation of lifestyle-related health risks in the study population (18, 19).

### Measurement of infertility

The primary outcome variable of this study was infertility. Infertility status was determined based on self-reported data from women who participated in the NHANES Reproductive Health Questionnaire (RHQ). Specifically, the response to item RHQ074, “Have you ever attempted to become pregnant over a period of at least a year without becoming pregnant?” was used to classify women. Those who answered “yes” to this question were categorized as having infertility (20).

### Covariables

The collection of variables in this study includes demographic characteristics such as age, BMI, race, marital status, education level, poverty-income ratio (PIR), history of ever pregnancies. Comorbid conditions such as diabetes, hypertension, metabolic syndrome, as well as laboratory biochemical indicators like estimated glomerular filtration rate (eGFR), HDL cholesterol, HbA1c, and albumin.

Diabetes mellitus was identified based on a combination of factors: a hemoglobin A1c (HbA1c) concentration of 6.5% or greater, a fasting plasma glucose (FPG) level exceeding 126 mg/dL, documented use of hypoglycemic agents, or a self-reported medical history of diabetes. Hypertension was diagnosed when systolic and diastolic blood pressure measurements reached or surpassed 140/90 mmHg, patients were on antihypertensive therapy, or there was a self-reported history of hypertension. For the identification of metabolic syndrome in the adult population, the criteria established by the National Cholesterol Education Program’s Adult Treatment Panel III (NCEP-ATP III) (21, 22) were applied. These criteria provide a standardized approach for the clinical assessment and diagnosis of these conditions.

### Statistical analysis

Data processing in our study adhered to NHANES analysis guidelines, ensuring that no variable had missing data exceeding 10%. All analyses incorporated appropriate sampling weights. Continuous variables were presented as weighted means accompanied by standard errors, while categorical variables were depicted as weighted percentages with their respective standard errors. Disparities in continuous baseline attributes were assessed using Student’s t-test, and for categorical variables, the chi-square test was applied. A weighted multiple logistic regression model was utilized to investigate the association between OBS and infertility, adjusting for various demographic and health-related variables such as age, race, marital status, educational attainment, PIR, DM, hypertension, MetS, eGFR, HDL cholesterol, HbA1c, and albumin. OBS was categorized into quartiles for trend analysis and subgroup analyses were conducted to explore relationships within different demographic and health characteristic strata. Interaction tests were performed to assess consistency across subgroups. Nonlinear relationships were examined using RCS and multiple imputation techniques were employed for reliability. Statistical analyses were carried out using R software (version 4.3.0) and Free Statistics software (version 1.9.2).

## Results

### Baseline characteristics

**Figure 1:**
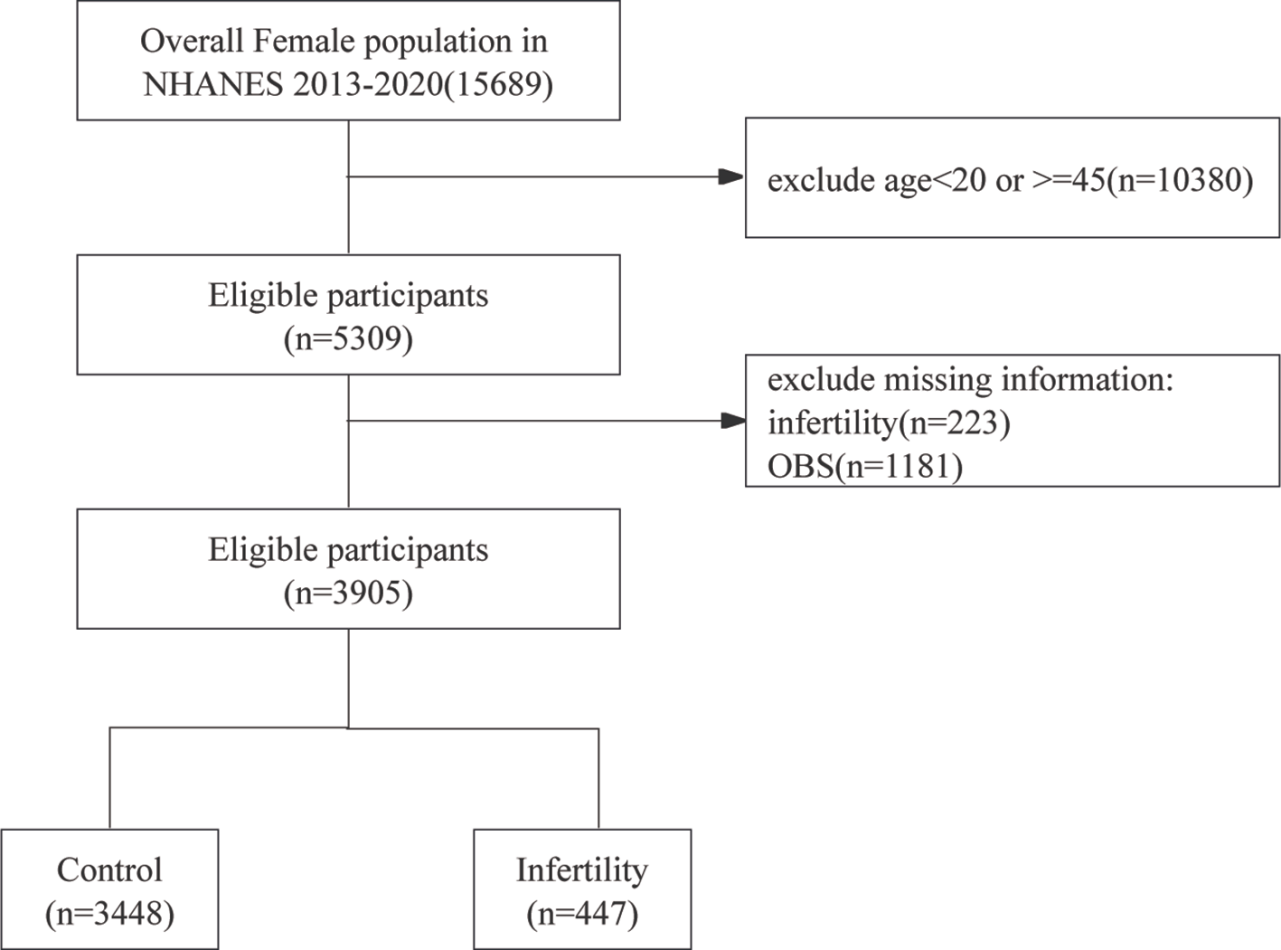
Flow chart of participants selection. NHANES, National Health and Nutrition Examination Survey.

**Figure 1** shows a flowchart of the data integration procedure. **Table 1** presents the initial demographic and characteristics of the participants in the study, stratified by the presence of infertility. Participants with infertility had significantly lower scores in the OBS, OBS dietary, and OBS lifestyle categories compared to their fertile counterparts. Infertile participants showed distinct characteristics, such as older age and a higher prevalence of prior pregnancies. Furthermore, this group had a higher occurrence of comorbidities like diabetes mellitus, hypertension, and metabolic syndrome. On the other hand, individuals in the infertile group had lower levels of high-density lipoprotein (HDL) cholesterol and albumin compared to fertile individuals. These results indicate an intricate relationship between fertility status and different health factors.

**Table 1:** Baseline characteristics of participants, weighted(N=3905).

Variable	Total	Fertility	Infertility	P value
OBS	21.22 ± 0.20	21.42 ± 0.20	19.74 ± 0.37	< 0.0001
OBS.dietary	16.99 ± 0.18	17.12 ± 0.18	15.98 ± 0.33	< 0.001
OBS.lifestyle	4.23 ± 0.05	4.29 ± 0.05	3.76 ± 0.11	< 0.0001
Age(%)				< 0.0001
<35 year	59.98(0.02)	61.68(1.22)	47.49(3.31)	
>=35 year	40.02(0.02)	38.32(1.22)	52.51(3.31)	
Race (%)				0.28
Non-Hispanic White	56.06(0.03)	55.48(2.13)	60.33(3.40)	
Non-Hispanic Black	13.74(0.01)	13.85(1.22)	12.97(1.56)	
Mexican Ameirican	11.73(0.01)	11.83(1.19)	10.95(1.82)	
Other Race	18.47(0.01)	18.84(1.19)	15.75(2.03)	
Marital status(%)				< 0.0001
Solitude	38.26(0.02)	48.83(1.45)	26.00(2.90)	
Cohabitation	44.56(0.02)	51.17(1.45)	74.00(2.90)	
Education level(%)				0.17
Less than or high school	29.99(0.01)	29.64(1.51)	32.63(2.35)	
Above high school	69.99(0.03)	70.36(1.51)	67.37(2.35)	
PIR(%)				0.23
<1.3	25.68(0.01)	28.03(1.20)	24.14(2.45)	
13.,3.5	33.67(0.02)	35.61(1.24)	39.86(2.99)	
>=3.5	33.85(0.02)	36.36(1.67)	36.00(2.98)	
Ever pregnant(%)				< 0.0001
No	31.45(0.02)	33.63(1.28)	15.53(1.85)	
Yes	68.49(0.02)	66.37(1.28)	84.47(1.85)	
DM(%)				0.001
No	85.11(0.03)	90.04(0.61)	84.84(1.70)	
Pre DM	4.20(0.00)	4.37(0.47)	4.74(1.15)	
DM	5.86(0.00)	5.59(0.41)	10.42(1.49)	
Hypertension(%)				0.004
No	86.47(0.03)	87.21(0.77)	81.25(2.19)	
Yes	13.49(0.01)	12.79(0.77)	18.75(2.19)	
MetS(%)				< 0.001
No	75.00(0.03)	80.05(1.11)	69.41(2.97)	
Yes	20.17(0.01)	19.95(1.11)	30.59(2.97)	
eGFR	111.06 ± 0.62	111.31 ± 0.64	109.26 ± 1.14	0.07
HDL(mmol.L)	1.48 ± 0.01	1.49 ± 0.01	1.39 ± 0.03	0.001
HbA1c	5.33 ± 0.01	5.31 ± 0.01	5.48 ± 0.03	< 0.0001
Albumin(g.L)	41.05 ± 0.10	41.10 ± 0.10	40.64 ± 0.24	0.07

Values are mean +/- SD (continuous variables) or n% (categorical variables) are weighted.

OBS, Oxidative balance score; BMI, Body mass index; PIR, Poverty to income ratio; DM, Diabetes mellitus; MetS, Metabolic syndrome; eGFR, estimated Glomerular Filtration Rate.

### Association between oxidative balance score and infertility

**Table 2** presents the results of a weighted regression analysis that examines the relationship between the OBS and the prevalence of infertility. The analysis revealed that for each one-unit increase in the OBS, there was a corresponding decrease in the likelihood of experiencing infertility, with an OR of 0.97 and a 95%CI of 0.95 to 0.98. These findings suggest a small but statistically significant protective impact of higher OBS against infertility. This association was consistent across different models, with the OR remaining stable. In the fully adjusted model, an increment of one point in OBS corresponded to a 3% decrease in the likelihood of infertility (OR: 0.97, 95% CI: 0.95, 0.99).

**Table 2:** Association between OBS with infertility.

Character	Crude model (95%CI)	Model 1 (95%CI)	Model 2 (95%CI)	Model 3 (95%CI)
OBS	0.97 (0.95,0.98)*	0.96 (0.94,0.98)*	0.97 (0.95,0.99)*	0.97 (0.95,0.99)*
OBS quantile				
Q1	ref	ref	ref	ref
Q2	0.70 (0.54,0.91)*	0.77 (0.55,1.06)	0.74 (0.53,1.02)	0.76 (0.54,1.07)
Q3	0.70 (0.51,0.96)*	0.71 (0.48,1.05)	0.74 (0.50,1.11)	0.80 (0.53,1.21)
Q4	0.42 (0.30,0.59)*	0.40 (0.27,0.60)*	0.45 (0.29,0.69)*	0.47 (0.31,0.72)*
p for trend	<0.0001	<0.0001	<0.001	0.001
OBS lifestyle	0.82 (0.76,0.89)*	0.82 (0.74,0.91)*	0.85 (0.76,0.95)*	0.85 (0.75,0.96)*
OBS lifestyle quantile				
Q1	ref	ref	ref	ref
Q2	0.80 (0.56,1.13)	0.76 (0.50,1.14)	0.79 (0.53,1.17)	0.82 (0.53,1.25)
Q3	0.61 (0.43,0.86)*	0.58 (0.38,0.90)*	0.65 (0.42,1.00)*	0.64 (0.41,1.01)
Q4	0.36 (0.21,0.60)*	0.38 (0.21,0.71)*	0.44 (0.23,0.85)*	0.45 (0.23,0.88)*
p for trend	<0.0001	<0.001	0.01	0.01
OBS dietary	0.97 (0.96,0.99)*	0.97 (0.95,0.99)*	0.97 (0.95,0.99)*	0.98 (0.96,0.99)*
OBS dietary quantile				
Q1	ref	ref	ref	ref
Q2	0.63 (0.48,0.81)*	0.67 (0.48,0.93)*	0.68 (0.49,0.95)*	0.68 (0.49,0.95)*
Q3	0.84 (0.64,1.11)	0.86 (0.60,1.21)	0.85 (0.59,1.22)	0.90 (0.62,1.32)
Q4	0.50 (0.35,0.69)*	0.46 (0.31,0.70)*	0.51 (0.33,0.78)*	0.52 (0.35,0.80)*
p for trend	<0.001	0.002	0.01	0.01

Crude model: no covariates were adjusted.

Model 1, age, marital status, race, education level, PIR and ever pregnant were adjusted.

Model 2, Model 1+DM and hypertension were adjusted.

Model 3, Model 2+MetS, eGFR, alumin, HDL and HbA1c were adjusted.

*mean p <0.05.

When OBS was modeled as a quadratic variable, the unadjusted model showed that, compared to the first quartile, the adjusted ORs for the second, third, and fourth quartiles were 0.7 (95% CI: 0.54-0.91), 0.7 (95% CI: 0.51-0.96), and 0.42 (95% CI: 0.30-0.59), respectively. After adjusting for relevant covariates, the analysis found no statistically significant disparities between the second (Q2) and third (Q3) quartiles compared to the first quartile (Q1). However, the fourth quartile (Q4) displayed a significant decrease (Model 3 adjusted OR: 0.47, 95% CI: 0.31-0.72), suggesting a linear trend for OBS in relation to infertility diagnosis (trend p < 0.05).

Additionally, we examined the distinct relationships between OBS dietary and OBS lifestyle components with infertility. The data in **Table 2** indicate that both OBS dietary and OBS lifestyle components are significantly correlated with infertility in both unadjusted and adjusted models.

### Subgroup analyses

Subgroup and interaction analyses were performed, stratified by age, diabetes, hypertension, history of previous pregnancies, and metabolic syndrome, to evaluate the relationship between the OBS and infertility and to identify potential modifiers of this relationship. **Table 3** indicates no statistically significant differences in the associations with age, diabetes, hypertension, or metabolic syndrome. However, a significant interaction was observed for the history of previous pregnancies (interaction p < 0.05).

**Table 3:** Subgroup analysis between OBS with infertility.

Character	Crude model (95%CI)	Model 1 (95%CI)	Model 2 (95%CI)	Model 3 (95%CI)	p for interaction
Age					0.48
<35	0.95 (0.93,0.98)	0.96 (0.93,0.99)	0.96 (0.93,0.99)	0.96 (0.93,0.99)	
>=35	0.98 (0.95,1.00)	0.97 (0.94,1.00)	0.97 (0.94,1.00)	0.97 (0.95,1.00)	
DM					0.9
No	0.97 (0.95,0.99)	0.97 (0.94,0.99)	0.97 (0.95,0.99)	0.97 (0.95,0.99)	
PreDM	0.92 (0.85,1.00)	0.91 (0.80, 1.03)	0.91 (0.83, 1.01)	0.91 (0.82, 1.02)	
DM	1.00 (0.94,1.06)	0.98 (0.89, 1.08)	0.98 (0.88, 1.08)	1.00 (0.89, 1.11)	
Hypertension					0.99
No	0.97 (0.95,0.98)	0.96 (0.94,0.98)	0.97 (0.95,0.99)	0.97 (0.95,1.00)	
Yes	0.97 (0.94,1.01)	0.96 (0.92,1.01)	0.96 (0.91, 1.01)	0.96 (0.92,1.01)	
Ever pregnant					0.01
No	0.92 (0.88,0.95)	0.93 (0.89, 0.98)	0.93 (0.88, 0.98)	0.95 (0.89, 1.01)	
Yes	0.98 (0.97,0.99)	0.97 (0.95,0.99)	0.98 (0.96,1.00)	0.98 (0.96,1.00)	
MetS_ATP					0.47
No	0.97 (0.95,0.99)	0.97 (0.95,1.00)	0.97 (0.95,1.00)	0.97 (0.95,1.00)	
Yes	0.96 (0.92,0.99)	0.96 (0.92,1.00)	0.96 (0.92,1.00)	0.96 (0.92,1.00)	

Crude model: no covariates were adjusted.

Model 1, age, marital status, race, education level, PIR and ever pregnant were adjusted.

Model 2, Model 1+DM and hypertension were adjusted.

Model 3, Model 2+MetS, eGFR, alumin, HDL and HbA1c were adjusted.

*mean p <0.05.

Among individuals without a history of previous pregnancies, OBS showed a stronger negative correlation with infertility [OR 0.95, 95% CI (0.89, 1.01)] than among those with such a history [OR 0.98, 95% CI (0.96, 1)]. A significant interaction between age and history of previous pregnancies was noted in the OBS lifestyle component (interaction p < 0.05) (**Table 4**). For individuals under 35 years, OBS demonstrated a significantly stronger negative correlation with infertility [OR 0.75, 95% CI (0.65, 0.85)] compared to those aged 35 and above [OR 0.98, 95% CI (0.84, 1.16)]. Additionally, in individuals without a history of previous pregnancies, OBS lifestyle had a notably stronger negative correlation with infertility [OR 0.63, 95% CI (0.48, 0.83)] than among those with a history [OR 0.92, 95% CI (0.82, 1.05)]. In the case of OBS dietary (**Table 5**), no interaction was observed, yet individuals without a history of previous pregnancies showed a slightly stronger negative association with infertility [OR 0.97, 95% CI (0.91, 1.03)] compared to those with a history [OR 0.98, 95% CI (0.96, 1)].

**Table 4:** Subgroup analysis between OBS lifestyle with infertility.

Character	Crude model (95%CI)	Model 1 (95%CI)	Model 2 (95%CI)	Model 3 (95%CI)	p for interaction
Age					< 0.001
<35	0.74 (0.67,0.82)	0.73 (0.65,0.83)	0.74 (0.64,0.84)	0.75 (0.65,0.85)	
>=35	0.94 (0.83,1.06)	0.92 (0.78,1.09)	1.00 (0.86,1.16)	0.98 (0.84,1.16)	
DM					0.15
No	0.83 (0.75,0.91)	0.83 (0.74,0.94)	0.84 (0.74,0.94)	0.82 (0.73,0.93)	
PreDM	0.96 (0.64,1.44)	0.77 (0.45, 1.31)	0.91 (0.50, 1.66)	0.97 (0.44, 2.16)	
DM	1.09 (0.88,1.36)	1.07 (0.72, 1.60)	1.06 (0.70, 1.62)	1.08 (0.71, 1.63)	
Hypertension					0.3
No	0.81 (0.75,0.89)	0.81 (0.73,0.91)	0.84 (0.75,0.94)	0.83 (0.74,0.94)	
Yes	0.94 (0.77,1.14)	0.93 (0.73,1.19)	0.95 (0.73, 1.24)	0.97 (0.74,1.27)	
Ever pregnant					< 0.001
No	0.64 (0.54,0.75)	0.63 (0.51, 0.78)	0.65 (0.53, 0.80)	0.63 (0.48, 0.83)	
Yes	0.90 (0.82,1.00)	0.88 (0.79,0.99)	0.93 (0.83,1.04)	0.92 (0.82,1.05)	
MetS_ATP					0.08
No	0.85 (0.77,0.93)	0.83 (0.74,0.94)	0.83 (0.74,0.94)	0.79 (0.69,0.90)	
Yes	0.92 (0.79,1.08)	0.97 (0.80,1.18)	0.98 (0.81,1.19)	1.01 (0.84,1.22)	

Crude model: no covariates were adjusted.

Model 1, age, marital status, race, education level, PIR and ever pregnant were adjusted.

Model 2, Model 1+DM and hypertension were adjusted.

Model 3, Model 2+MetS, eGFR, alumin, HDL and HbA1c were adjusted.

*mean p <0.05.

**Table 5:** Subgroup analysis between OBS dietary with infertility.

Character	Crude model (95%CI)	Model 1 (95%CI)	Model 2 (95%CI)	Model 3 (95%CI)	p for interaction
Age					0.94
<35	0.97 (0.94,0.99)	0.97 (0.94,1.00)	0.98 (0.95,1.01)	0.97 (0.94,1.01)	
>=35	0.98 (0.95,1.00)	0.97 (0.94,1.00)	0.97 (0.94,1.00)	0.97 (0.94,1.00)	
DM					0.9
No	0.97 (0.95,0.99)	0.97 (0.95,1.00)	0.97 (0.95,1.00)	0.98 (0.96,1.00)	
PreDM	0.92 (0.84,1.01)	0.92 (0.81, 1.04)	0.92 (0.82, 1.02)	0.92 (0.82, 1.02)	
DM	0.99 (0.93,1.06)	0.98 (0.88, 1.08)	0.97 (0.88, 1.08)	0.99 (0.89, 1.11)	
Hypertension					0.78
No	0.97 (0.96,0.99)	0.97 (0.95,0.99)	0.97 (0.95,1.00)	0.98 (0.95,1.00)	
Yes	0.98 (0.94,1.01)	0.96 (0.92,1.01)	0.96 (0.91, 1.01)	0.96 (0.92, 1.01)	
Ever pregnant					0.14
No	0.93 (0.89,0.98)	0.95 (0.91, 1.01)	0.95 (0.90, 1.01)	0.97 (0.91, 1.03)	
Yes	0.98 (0.97,1.00)	0.97 (0.96,0.99)	0.98 (0.96,1.00)	0.98 (0.96,1.00)	
MetS_ATP					0.26
No	0.98 (0.96,1.00)	0.98 (0.95,1.01)	0.98 (0.95,1.01)	0.98 (0.96,1.01)	
Yes	0.96 (0.92,1.00)	0.95 (0.91,1.00)	0.96 (0.92,1.00)	0.96 (0.91,1.00)	

Crude model: no covariates were adjusted.

Model 1, age, marital status, race, education level, PIR and ever pregnant were adjusted.

Model 2, Model 1+DM and hypertension were adjusted.

Model 3, Model 2+MetS, eGFR, alumin, HDL and HbA1c were adjusted.

*mean p <0.05.

**Figure 2:**
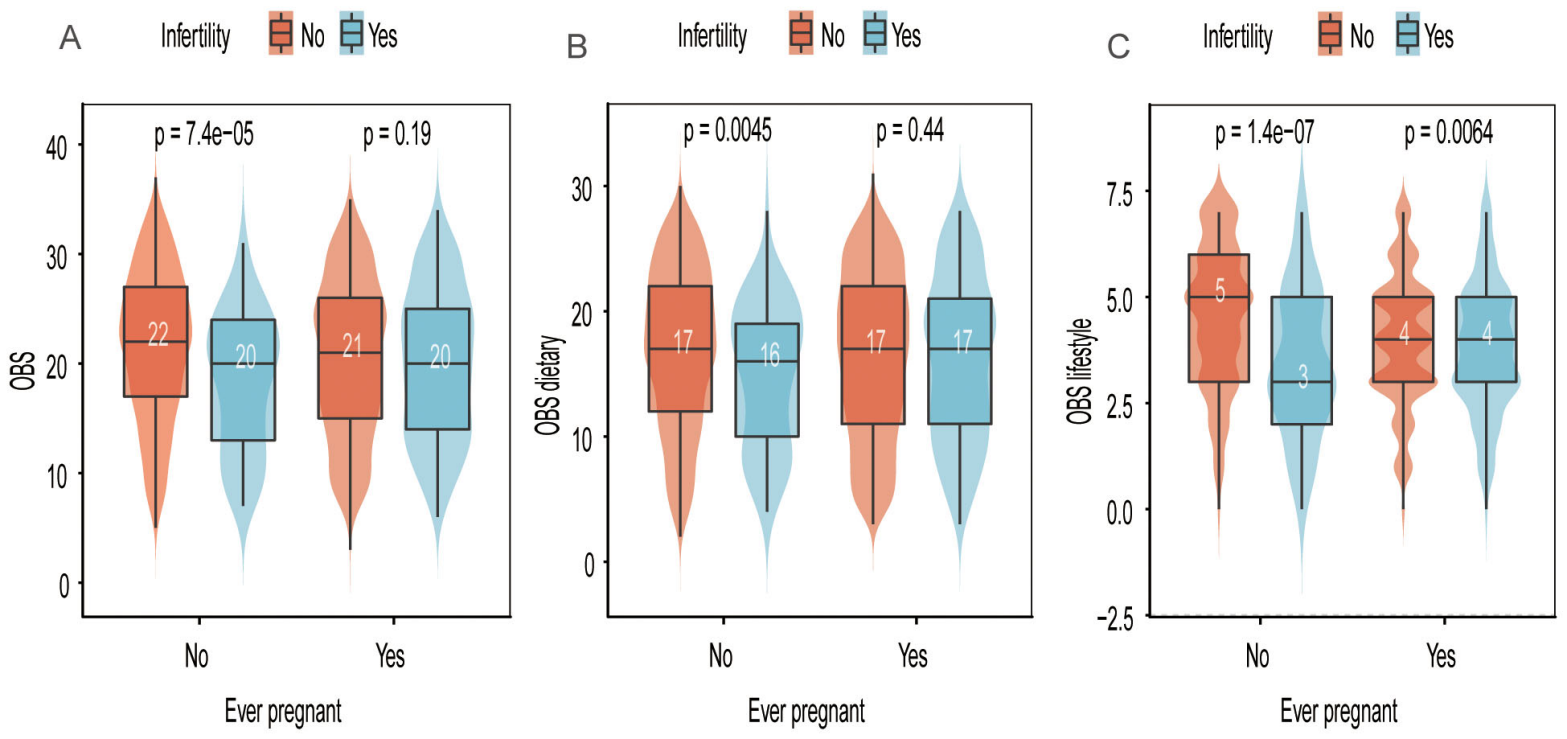
Distribution of OBS, OBS dietary, and OBS lifestyle scores in relation to “ever pregnant” and infertility status. **(A)** OBS scores stratified by infertility and pregnancy history. **(B)** OBS dietary scores stratified by infertility and pregnancy history. **(C)** OBS lifestyle scores stratified by infertility and pregnancy history.

**Figure 2** illustrates that OBS was significantly higher in individuals without a history of previous pregnancies compared to the infertile population, a difference not observed in those with a history of previous pregnancies. Similar findings were noted for OBS dietary.

### Restricted cubic spline analysis

**Figure 3:**
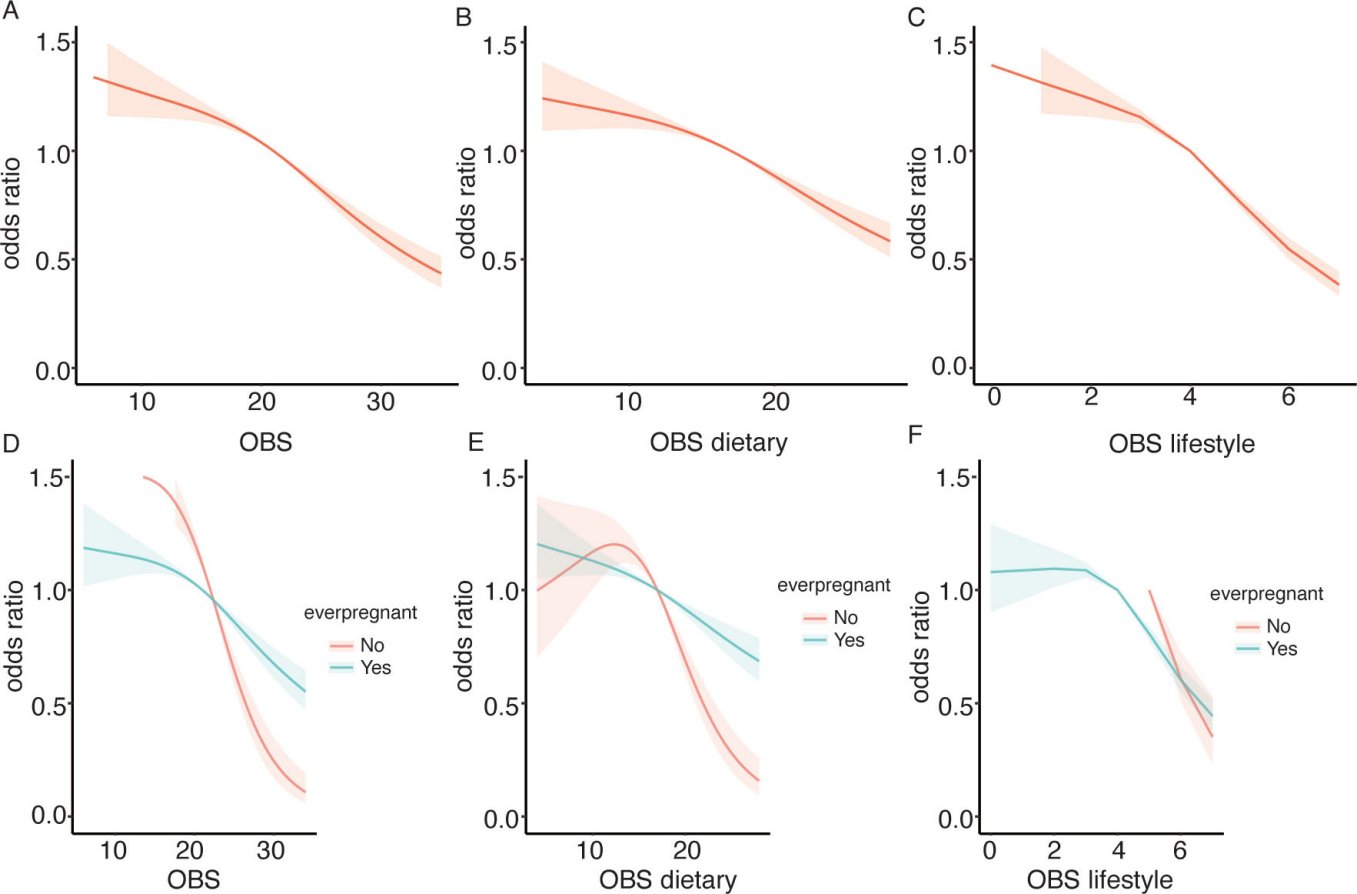
Displays the association between the OBS and infertility risk using RCS. **(A)** for the overall OBS, **(B)** for the dietary component, and **(C)** for the lifestyle component. Stratified analyses by ever pregnant status are presented in **(D–F)**, with adjustments for confounding variables.

To investigate the potential non-linear association between the OBS and infertility, we performed RCS analysis. Employing a logistic regression model with smooth curve fitting, after controlling for additional factors, we found that the non-linear connection between OBS and infertility was not statistically significant. (**Figure 3**). Additionally, the non-linear associations between OBS dietary, OBS lifestyle components, and infertility were also found to be non-significant.

### Sensitivity analysis

**Supplementary Table 2** presents the findings from a re-analysis of the raw data without multiple imputing missing values, which corroborates the main study findings. Regardless of how OBS was considered - as either a continuous or categorical variable - a significant negative correlation with infertility was observed.

## Discussion

Our analysis revealed a significant negative correlation between the OBS and female infertility. Specifically, we found that higher OBS levels were associated with a decreased incidence of infertility, with each one-point increase in OBS corresponding to a 3% reduction in infertility risk. This relationship remained consistent across multiple adjusted models, suggesting a robust association independent of demographic and clinical confounders.

The subgroup analyses yielded particularly insightful findings. The protective effect of OBS was significantly more pronounced among women under 35 years compared to their older counterparts. While the total OBS showed similar trends across age groups, the lifestyle component of OBS demonstrated a significant interaction with age, with younger women experiencing a substantially stronger protective effect (25% risk reduction per point) than women aged 35 and older. This age-dependent relationship reflects the differing etiologies of infertility across age groups. In younger women, conditions potentially more responsive to oxidative balance modulation—such as polycystic ovary syndrome (PCOS), endometriosis, and unexplained infertility—predominate. Oxidative stress in PCOS affects ovarian follicles, disrupting their development and maturation, which can impair fertility (23–25). Similarly, oxidative stress is implicated in endometriosis and unexplained infertility, leading to poor endometrial receptivity and negatively affecting pregnancy outcomes (26–28). Conversely, in women over 35, age-related decline in ovarian reserve becomes the primary driver of infertility, a process less amenable to improvement through oxidative balance enhancement.

Younger women benefit more from lifestyle interventions that enhance oxidative balance due to their greater biological plasticity and antioxidant capacity. Studies demonstrate that while older women can improve oxidative stress markers through resistance training, the improvements are more substantial in younger women due to their naturally higher baseline antioxidant capacity (29–31). The biological mechanisms underlying this age-dependent response involve multiple pathways. Younger women possess more robust antioxidant defense systems, greater mitochondrial efficiency, and enhanced cellular repair mechanisms (2, 32). Oxidative stress negatively affects reproductive processes by causing DNA damage and mitochondrial dysfunction, which are more pronounced in older women, while younger women are better equipped to manage oxidative stress due to more efficient cellular repair mechanisms (33–35). Antioxidants play a critical role in protecting oocytes from oxidative damage, and younger women benefit more from these protective mechanisms, which help maintain oocyte quality and fertility (36, 37).

Another key finding was the differential impact of OBS based on previous pregnancy history. The protective association between OBS and infertility was significantly stronger in women with primary infertility compared to those with secondary infertility. This suggests that women who have never achieved pregnancy represent a distinct population in which oxidative stress plays a more prominent pathophysiological role. Primary infertility may involve more significant underlying oxidative-mediated pathologies that could potentially be mitigated through antioxidant-rich diets and lifestyle modifications. Conversely, women with secondary infertility may have different etiologies less responsive to oxidative balance modulation.

Women with primary infertility exhibit significantly higher levels of oxidative stress compared to fertile women, with elevated levels of malondialdehyde (MDA), a marker of lipid peroxidation (28). This finding is consistent across various studies (38, 39), highlighting the role of oxidative stress in infertility. While data on secondary infertility are limited, the general impact of oxidative stress on reproductive health suggests it plays a role in both conditions, though potentially through different mechanisms or to varying degrees.

These findings align with our understanding of the mechanistic role of oxidative stress in female reproduction. Physiological levels of reactive oxygen species (ROS) are essential for normal follicular development, ovulation, and fertilization (40–42). However, excessive ROS production can damage cellular components, impair mitochondrial function, and trigger apoptotic pathways in reproductive tissues. Low ROS levels in follicular fluid correlate with successful pregnancy outcomes in IVF treatments (43), suggesting a potential prognostic indicator for IVF efficacy. Our study extends these mechanistic insights by demonstrating that a comprehensive measure of oxidative balance correlates with fertility outcomes at the population level, particularly in specific subgroups of women.

The individual components of OBS provide further insights into potential intervention strategies. Dietary antioxidants, including vitamins C and E, protect reproductive cells against oxidative damage.

Amini et al (44) demonstrated that supplementation with vitamins C and E can reduce serum ROS levels compared to a control group, resulting in increased antioxidant concentrations in serum and follicular fluid. This leads to enhanced oocyte and IVF embryo quality, as well as improved pregnancy outcomes (45). Research (46) from the NHANES indicated a negative correlation between dietary fiber intake and female infertility. Li et al (47) found that Erastin, an inducer of ferroptosis, elevated ROS levels in mice under iron accumulation conditions, potentially triggering cell death mechanisms that could alleviate endometriosis and improve fertility. A Nigerian study (48) comparing serum metal levels in women with unexplained infertility to those with normal fertility revealed lower copper, zinc, and selenium levels in the infertile group. These micronutrients serve as essential cofactors for antioxidant enzymes, and their deficiency may compromise oxidative defense mechanisms in reproductive tissues.

Lifestyle factors within the OBS showed particularly strong associations with fertility outcomes. Smoking, for instance, induces oxidative stress in reproductive tissues, with women who smoke exhibiting lower success rates in assisted reproductive technologies (ART). Smoking increases ROS levels and reduces antioxidant defenses, affecting both sperm and oocyte quality (28, 49, 50). Meta-analyses show that smoking decreases live birth rates and clinical pregnancy rates per ART cycle, while increasing miscarriage rates. These effects are significant and highlight the detrimental impact of smoking on ART success (51–53).

Alcohol consumption similarly promotes oxidative stress through its metabolism, with even moderate intake potentially affecting fertility outcomes (28, 54). Obesity, another pro-oxidant factor in the OBS, is associated with both systemic inflammation and oxidative stress, contributing to ovulatory dysfunction and decreased endometrial receptivity (55–57). A cohort study by Jose Bellver et al (58) indicates that obesity may impair endometrial receptivity, contributing to infertility and negative outcomes in ART.

Our study has several methodological strengths. The use of a nationally representative NHANES population enhances generalizability, while the comprehensive adjustment for confounding variables increases confidence in the observed associations. Furthermore, our approach, which examines both total OBS and its components, provides greater insight into the relative contributions of dietary and lifestyle factors to fertility outcomes.

Nevertheless, this research has limitations that warrant consideration. The cross-sectional design precludes establishment of causality, and the reliance on self-reported infertility data may introduce recall bias. Additionally, while we adjusted for numerous potential confounders, residual confounding cannot be entirely ruled out. The NHANES database lacks comprehensive data on environmental exposures, such as air pollution, pesticides, and endocrine-disrupting chemicals, which may influence both oxidative stress and fertility.

In conclusion, our findings demonstrate that higher OBS is associated with lower infertility risk, with particularly strong effects observed in younger women and those with primary infertility. These results suggest that optimizing oxidative balance through dietary and lifestyle modifications may represent an effective strategy for reducing infertility risk in specific populations. Future longitudinal studies and clinical trials are needed to establish causality and determine whether targeted interventions to improve oxidative balance can enhance fertility outcomes in women attempting conception

## Data Availability

Publicly available datasets were analyzed in this study. This data can be found here: https://wwwn.cdc.gov/nchs/nhanes/search/default.aspx.

## Supplementary material

Supplementary Table 1 Oxidative balance score assignment scheme.

Supplementary Table 2 Sensitivity Analysis of the Association between OBS and Infertility after Multiple Imputation Crude model: no covariates were adjusted. Model 1, age, marital status, race, education level, PIR and ever pregnant were adjusted. Model 2, Model 1+DM and hypertension were adjusted. Model 3, Model 2+MetS, eGFR, alumin, HDL and HbA1c were adjusted. * mean p <0.05.
