# Supplementary material for: The role of oxidative balance lifestyle factors in reducing female infertility risk: insights from a population-based study
Source: Front Endocrinol (Lausanne). 2025 Jun 19;16:1444832. doi: 10.3389/fendo.2025.1444832 (PMC12221921; doi:10.3389/fendo.2025.1444832)
Supplement: Supplementary Table 1 — Oxidative balance score assignment scheme. [file SupplementaryFile1.docx]

**Supplement tables**

Supplement Table 1. Oxidative balance score assignment scheme.

| OBS components | Property | Scoring assignment | | |
| --- | --- | --- | --- | --- |
|  |  | 0 | 1 | 2 |
| Dietary components |  |  |  |  |
| Dietary fiber (g/d) | Antioxidant | Tertile 1 | Tertile 2 | Tertile 3 |
| Carotene (RE/d) | Antioxidant | Tertile 1 | Tertile 2 | Tertile 3 |
| Riboflavin (mg/d) | Antioxidant | Tertile 1 | Tertile 2 | Tertile 3 |
| Niacin (mg/d) | Antioxidant | Tertile 1 | Tertile 2 | Tertile 3 |
| Vitamin B6 (mg/d) | Antioxidant | Tertile 1 | Tertile 2 | Tertile 3 |
| Total folate (mcg/d) | Antioxidant | Tertile 1 | Tertile 2 | Tertile 3 |
| Vitamin B12 (mcg/d) | Antioxidant | Tertile 1 | Tertile 2 | Tertile 3 |
| Vitamin C (mg/d) | Antioxidant | Tertile 1 | Tertile 2 | Tertile 3 |
| Vitamin E (ATE) (mg/d) | Antioxidant | Tertile 1 | Tertile 2 | Tertile 3 |
| Calcium (mg/d) | Antioxidant | Tertile 1 | Tertile 2 | Tertile 3 |
| Magnesium (mg/d) | Antioxidant | Tertile 1 | Tertile 2 | Tertile 3 |
| Zinc (mg/d) | Antioxidant | Tertile 1 | Tertile 2 | Tertile 3 |
| Copper (mg/d) | Antioxidant | Tertile 1 | Tertile 2 | Tertile 3 |
| Selenium (mcg/d) | Antioxidant | Tertile 1 | Tertile 2 | Tertile 3 |
| Total fat (g/d) | Prooxidant | Tertile 1 | Tertile 2 | Tertile 3 |
| Iron (mg/d) | Prooxidant | Tertile 1 | Tertile 2 | Tertile 3 |
| Lifestyle components |  |  |  |  |
| Physical activity(MET-minute/week) | Antioxidant | <400 | 400–1,000 | >1,000 |
| Alcohol (g/d) | Prooxidant | >30 | 0–30 | None |
| Body mass index (kg/m2) | Prooxidant | >30 | 25–30 | <25 |
| Cotinine (ng/mL) | Prooxidant | >0.038 | 0.038–1.13 | <1.13 |

Supplement Table 2. Sensitivity Analysis of the Association between OBS and Infertility after Multiple Imputation

| Character | crude model(95%CI) | Model 1(95%CI) | Model 2(95%CI) | Model 3(95%CI) |
| --- | --- | --- | --- | --- |
| OBS | 0.97(0.95,0.98)* | 0.96(0.95,0.98)* | 0.96(0.95,0.98)* | 0.97(0.95,0.98)* |
| OBS quantile |  |  |  |  |
| Q1 | ref | ref | ref | ref |
| Q2 | 0.70(0.54,0.91)* | 0.72(0.55,0.95)* | 0.73(0.55,0.97)* | 0.73(0.55,0.98)* |
| Q3 | 0.70(0.51,0.96)* | 0.70(0.51,0.97)* | 0.71(0.51,0.99)* | 0.74(0.53,1.03) |
| Q4 | 0.42(0.30,0.59)* | 0.39(0.27,0.56)* | 0.39(0.27,0.57)* | 0.42(0.29,0.61)* |
| p for trend | <0.0001 | <0.0001 | <0.0001 | <0.0001 |
| OBS lifestyle | 0.82(0.76,0.89)* | 0.80(0.73,0.88)* | 0.81(0.74,0.89)* | 0.83(0.76,0.92)* |
| OBS lifestyle quantile |  |  |  |  |
| Q1 | ref | ref | ref | ref |
| Q2 | 0.80(0.56,1.13) | 0.71(0.50,1.00) | 0.72(0.51,1.02) | 0.77(0.53,1.10) |
| Q3 | 0.61(0.43,0.86)* | 0.56(0.39,0.81)* | 0.57(0.39,0.83)* | 0.60(0.41,0.89)* |
| Q4 | 0.36(0.21,0.60)* | 0.33(0.20,0.57)* | 0.35(0.20,0.60)* | 0.39(0.23,0.69)* |
| p for trend | <0.0001 | <0.0001 | <0.0001 | <0.0001 |
| OBS dietary |  |  |  |  |
| OBS dietary quantile | 0.97(0.96,0.99)* | 0.97(0.95,0.99)* | 0.97(0.95,0.99)* | 0.97(0.96,0.99)* |
| Q1 | ref | ref | ref | ref |
| Q2 | 0.63(0.48,0.81)* | 0.66(0.50,0.87)* | 0.67(0.51,0.88)* | 0.68(0.52,0.90)* |
| Q3 | 0.84(0.64,1.11) | 0.85(0.64,1.14) | 0.86(0.64,1.15) | 0.89(0.66,1.19) |
| Q4 | 0.50(0.35,0.69)* | 0.46(0.32,0.66)* | 0.46(0.32,0.66)* | 0.49(0.35,0.70)* |
| p for trend | <0.001 | <0.001 | <0.001 | <0.001 |

Crude model: no covariates were adjusted.

Model 1, age,marital status, race, education level, PIR and ever pregnant were adjusted.

Model 2, Model 1+DM and hypertension were adjusted.

Model 3, Model 2+MetS, eGFR, alumin, HDL and HbA1c were adjusted.

* mean p <0.05.
